# De novo transcriptome assembly of brackish water flea *Diaphanosoma celebensis* based on short-term cadmium and benzo[*a*]pyrene exposure experiments

**DOI:** 10.1186/s41065-018-0075-3

**Published:** 2018-11-17

**Authors:** Bo-Mi Kim, Seunghyun Kang, Ryeo-Ok Kim, Jee-Hyun Jung, Kyun-Woo Lee, Jae-Sung Rhee, Young-Mi Lee

**Affiliations:** 1 Unit of Polar Genomics, Korea Polar Research Institute, Incheon, 21990 South Korea; 2 Department of Life Science, College of Natural Sciences, Sangmyung University, Seoul, 03016 South Korea; 3 Oil and POPs Research Group, Korea Institute of Ocean Science and Technology, Geoje, 53201 South Korea; 4 Korea Institute of Ocean Science and Technology, 385, Haeyang-ro, Youngdo, Busan, 49111 South Korea; 5 Department of Marine Science, College of Natural Sciences, Incheon National University, Incheon, 22012 South Korea

**Keywords:** Benzo[*a*]pyrene, brackish water flea, Cadmium, *Diaphanosoma celebensis*, Transcriptome

## Abstract

To develop a brackish water flea as a promising model for marine monitoring, *Diaphanosoma celebensis* were exposed to two pollutants, cadmium (Cd) and benzo[*a*]pyrene (BaP), which have different chemical characteristics and distinct modes of metabolic action on aquatic animals. Twenty-four hours after exposure to Cd (2 mg/L) or BaP (25 μg/L), whole body transcriptomes were analyzed. In total, 99.6 Mbp were assembled from nine libraries, resulting in 98,458 transcripts with an N50 of 1883 bp and an average contig length of 968 bp. Functional gene annotations were performed using Gene Ontology, Eukaryotic Orthologous Groups, and Kyoto Encyclopedia of Genes and Genomes pathway analyses. Cd significantly modulated endocrine and digestive enzyme system. Following BaP treatment, DNA repair and circadian rhythm related metabolisms were significantly modulated. Both the chemicals induced stress response and detoxification metabolism. This brackish water flea genomic information will be useful to monitor estuaries and coastal regions, as water fleas have been confirmed as promising sentinel models in freshwater ecosystems.

**Electronic supplementary material:**

The online version of this article (10.1186/s41065-018-0075-3) contains supplementary material, which is available to authorized users.

## Background

Metals and polycyclic aromatic hydrocarbons (PAHs), which constitute a large portion of marine pollutants, are found worldwide in aquatic ecosystems due to their direct or indirect releases from sewage, industrial wastewater, oil spill, and mining [1, 2]. The pollutants have been detected in the coastal regions of South Korea [3, 4]. They have been causing increasing concerns to aquatic environment due to difficulty in biodegradation and potentials of bioaccumulation and biomagnification to higher trophic levels via food web [5]. Cd, a non-essential metal has been reported to adversely affect the physiology and biochemistry of aquatic invertebrates [6, 7]. Benzo[*a*]pyrene (BaP), PAH representative, adversely affects survival inhibition [8], growth inhibition [9], behavior impairment [10], and reproduction problems [11] of aquatic organisms. Since the sensitivity of signal pathway and subsequent detoxification cascade are very different for chemicals, we assume that transcriptome can differentially respond to certain chemical through specific and/or common responsive metabolism. Thus, we employed two distinct chemicals to detect early molecular markers and to validate whether the application of transcriptome profiling of brackish water flea is useful method for environmental risk assessment. Understanding the molecular effects of these pollutants may provide an alternative method to predict their toxicities.

Cladocera (crustacean) are filter-feeding planktonic water flea. *Daphnia* species have been extensively used due to their versatility as model animals for acute and/or chronic toxicity test and ecotoxicological application. Aquatic ecotoxicological studies mostly focus on freshwater *Daphnia* species such as *D. magna* or *D. pulex* (Crustacea, Cladocera, Daphniidae), while brackish water fleas are used sparingly. One of the advantages of freshwater *Daphnia* species is the availability of whole genome information and subsequent functional genomics application [12, 13]. There have been extensive transcriptome and genome studies on freshwater *Daphnia* species in the past decade. Estuaries and coastal regions are also important sites of ecotoxicology, with various land-derived contaminants. Sentinel organisms are useful for risk assessment and to ensuring safe and habitable environment in aquatic ecosystems. Thus, there is a need to develop robust model species for marine ecotoxicological research.

In general, most cladoceran appear to be restricted to freshwater, while *Diaphanosoma celebensis* (Crustacea, Cladocera, Sididae), is a euryhaline brackish water flea species distributes in tropical Asia [14]. In aquaculture industry, there is a need for developing tolerant or adapted cladoceran upon a wide range of salinity that can be applied from small aquaria to large-scale mass culture system under seawater condition. Changes in salinity have been considered as one of the crucial stressors, as even small change can directly affect osmoregulation and physiological homeostasis of marine animals. Since most aquaculture and fishery systems are located within coastal regions where the ultimate freshwater are released from inland, development of a species, which can maintain biological activity against steep salinity change, is important in aquaculture industry. They have been mainly studied in the field of aquaculture as a substitute live food [15, 16]. *D. celebensis* are primary consumers and are important for energy transfer to higher trophic levels in the aquatic food web.

*D. celebensis*, like freshwater *Daphnia* species, can be easily raised under laboratory culture conditions and require similar conditions with respect to feeding, water quality (pH), and light cycles, except for salinity and temperature. Furthermore, small size (adult 413–1112 μm), parthenogenetic mode of reproduction, short generation time (4–5 days), and easy maintenance in laboratory makes them suitable test organisms for marine ecotoxicological studies [17]. Genomics resource is very important for development of reliable model animal. Even though reference genomic database is absent in almost non-model animals, next generation sequencing (NGS) coupled with de novo assembly and appropriate bioinformatics tools enable us to use high quality genomic resource of certain animal.

Here, we developed a new transcriptomic resource of the brackish water flea *D. celebensis* using Illumina Hiseq 2500 platform and bioinformatics tools. To develop *D. celebensis* as a promising model animal for ecotoxicogenomics, following exposure to Cd and BaP, we analyzed the transcriptome and compared differentially expressed transcripts. The genomic information of *D. celebensis* will allow future investigation of molecular ecotoxicological pathways, with a particular focus on monitoring estuaries and coastal regions.

## Material and methods

### Culture and chemical exposure

The brackish water flea *D. celebensis* was obtained from Dr. Kyun-Woo Lee of the Korea Institute of Ocean Science and Technology and maintained in the Molecular Toxicology Laboratory of Sangmyung University since 2015 (Table 1). They were cultured in 0.2 μm-mesh filtered artificial seawater (Instant Ocean® Sea Salt, Instant Ocean, VA, USA), 15 practical salinity unit (psu), at 25 ± 1 °C under a 12 h: 12 h light/dark photoperiod. *Chlorella vularis* (4 × 10^7^ cells/L) cultured in Jaworski’s Medium was supplied as a food source thrice a week. All the chemicals and reagents used were of molecular biology grade and purchased from Sigma-Aldrich Co. (St. Louis, MO, USA) unless there is no description. For stock solution, Cd (as 2 mg/mL with CdCl_2_) and BaP (250 μg/mL) were prepared in sterile distilled water and dimethyl sulfoxide (DMSO, Sigma-Aldrich Co.), respectively. Final DMSO concentration was used as 0.01% that showed no significant difference between control and solvent control in preliminary gene expression study (data not shown). For transcriptome analysis, 350 *D. celebensis* adults (5 days old, mature female) were treated with Cd (2 mg/L, 350 μL from stock) and BaP (25 μg/L, 35 μL from stock) for 24 h, respectively, in 350 ml of media. Concentration of Cd applied was 1/10 of 24-h LC_10_ value (22.67 mg/L), and that of BaP was the highest concentration at which dead individuals were not observed in our preliminary test, based on results of [18]. Background and dissolved concentrations of both chemicals were analyzed using ICP-MP (Inductively Coupled Plasma – Mass Spectrometry (ICP-MS, PerkinElmer, NexION300, MA, USA) for Cd, and LC-MS for BaP. Background Cd concentration of media was measured as 0.001 mg/L, and BaP was not detected in the seawater. Dissolved concentrations of Cd and BaP were determined as 0.970 ± 0.002 mg/L and 25 μg/L, respectively.

**Table 1:** Characteristics of *Diaphanosoma celebensis* transcriptome sequencing project in compliance with the MIxS standard

Item	Description
Investigation type	Eukaryote transcriptome
Classification	Eukaryota; Opisthokonta; Metazoa; Eumetazoa; Bilateria; Protostomia; Ecdysozoa; Panarthropoda; Arthropoda; Mandibulata; Pancrustacea; Crustacea; Branchiopoda; Phyllopoda; Diplostraca; Cladocera; Ctenopoda; Sididae
Project name	*Diaphanosoma celebensis* transcriptome sequencing
Geographic location name	Malaysia coastal region (6° 30′ N, 103° 30′ E)
Maintenance	Korea Institute of Ocean Science and Technology
Provider	Dr. Kyun-Woo Lee
Environment (biome)	ENVO_00002030 (aquatic biome)
Environment (feature)	ENVO_01000321 (sea water environment)
Environment (material)	ENVO_00002150 (coastal sea water)
Tissue type	Whole body
Developmental stage	Adult
Sequencing method	Pyrosequencing
Sequencing platform	Illumina Hiseq 2500
Assembly	Trinity
Finishing strategy	Contig
Bioproject number	PRJNA464191
Data accessibility	GGQP00000000

### Illumina sequencing

A set of RNA samples for control and each chemical, comprised of three biological samples, was not pooled (i.e. total of 9 samples were prepared for total RNA preparation; three for control; three for Cd; three for BaP). Total RNA was extracted using TRIzol™ Reagent (Molecular Research Center, Inc., Cincinnati, OH, USA) according to the manufacturer’s instructions. DNA digestion was performed using DNase I (Sigma Aldrich, St Louis, MO, USA). Total RNA quantity (a NanoDrop® ND-8000 Spectrophotometer, Thermo Scientific, Wilmington, DE, USA) and quality by A230/260 and A260/280 ratios (Agilent 2100 Bioanalyzer, Agilent, Böblingen, Germany) were analyzed. Samples with RNA integrity number (> 7.5) were used for library preparation. Nine libraries were prepared using Tureseq™ stranded mRNA library prep Kit (Illumina, San Diego, CA, USA) at DNA link Inc. (Seoul, South Korea). Briefly, mRNA for each sample was purified and fragmented from total RNA (1 μg) by two rounds of purification using poly-T oligo-attached magnetic beads. Cleaved RNA fragments primed with random hexamers were reverse transcribed into first strand cDNA using reverse transcriptase, random primers, dUTP in place of dTTP. The products were purified and enriched with PCR to create the final strand specific cDNA library. The quality of amplified libraries was verified by capillary electrophoresis (Bioanalyzer, Agilent). After QPCR using SYBR Green PCR Master Mix (Applied Biosystems, Foster City, CA, USA), libraries that were index-tagged within a pool were combined in equimolar amounts. Cluster generation occurred in the flow cell of the cBot automated cluster generation system (Illumina). Finally, nine libraries of the three groups (i.e. control, Cd exposure, BaP exposure) were subjected to Illumina RNA sequencing (Additional file 1**:** Table S1). The sequenced *D. celebensis* cDNA libraries from each individual produced a large number of reads, at 56 to 78 million per library. The flow cell loaded on HIseq 2500 sequencing system (Illumina), performed sequencing with 2 × 100 bp read length. We trimmed the index and adaptor sequences using Trimmomatic [19] and removed low-quality reads using the FASTX toolkit [20] with the parameters set at -t, 20; −l, 70; and -Q, 33.

### De novo assembly and gene expression analysis

We assembled 99.6 Mbp (49% GC content) from nine libraries and removed low-quality reads (average quality score < 10), adapters, linkers, and PCR primers via quality filtering. For de novo transcriptome assembly, strand-specific mRNA reads from all nine samples were added to Trinity (https://github.com/trinityrnaseq/trinityrnaseq/wiki) as input to create a transcriptome. These assembled reads in FASTA format were added to CD-HIT-EST (http://weizhongli-lab.org/cd-hit) to remove redundant reads. Total trinity transcripts were 102,897 with an average length of 968 bp and an N50 length of 1883 bp (Additional file 1**:** Table S2). Finally, functional annotation of transcriptome was performed via Trinotate (https://trinotate.github.io) which includes a number of different methods such as BLASTX, BLASTP, HMMER, SignalP, TMHMM and RNAMMER. After removing redundant transcripts, total annotated transcripts were 98,458. For differentially expressed isoforms (DEI) analysis, abundance for each sample was estimated by Kallisto (https://pachterlab.github.io/kallisto) (Additional file 1**:** Table S3). To detect DEIs between sample 1 (control) and 2 (case), EdgeR (https://bioconductor.org/packages/release/bioc/html/edgeR.html) was performed, and three filtering processes were applied. Firstly, > 2 fold change was calculated and genes belonging to the following range were selected: Up-regulated: log2[case]-log2[control] > log2(2) =1; Downregulated: log2[case]-log2[control] < log2(1/2) = − 1. Secondly, genes with *p* value below 0.05 were selected. Lastly, genes less than 0.05 < FDR were filtered.

### Transcriptome annotation

Gene Ontology (GO) and Kyoto Encyclopedia of Genes and Genomes (KEGG) pathway analyses of all contigs were performed using Blast2GO sequence annotation tool (ver. 4.0) [21]. The specific GO composition of each category is presented as a Level 2 percentage. After aligning the contigs, we analyzed three principal categories (biological processes, cellular components, and molecular function) using default parameters. BLAST search and functional domain annotation by InterProScan of Blast2GO software package assigned 2194 contigs to at least one GO term (Additional file 1**:** Table S4). Finally, the assembled data was arranged in terms of read length, gene annotation, GenBank number, E-value, species, and species accession number. We calculated mRNA expression levels using reads per kilobase of transcriptome per million mapped reads (RPKM) method [22].

## Results and discussion

**Fig. 1:**
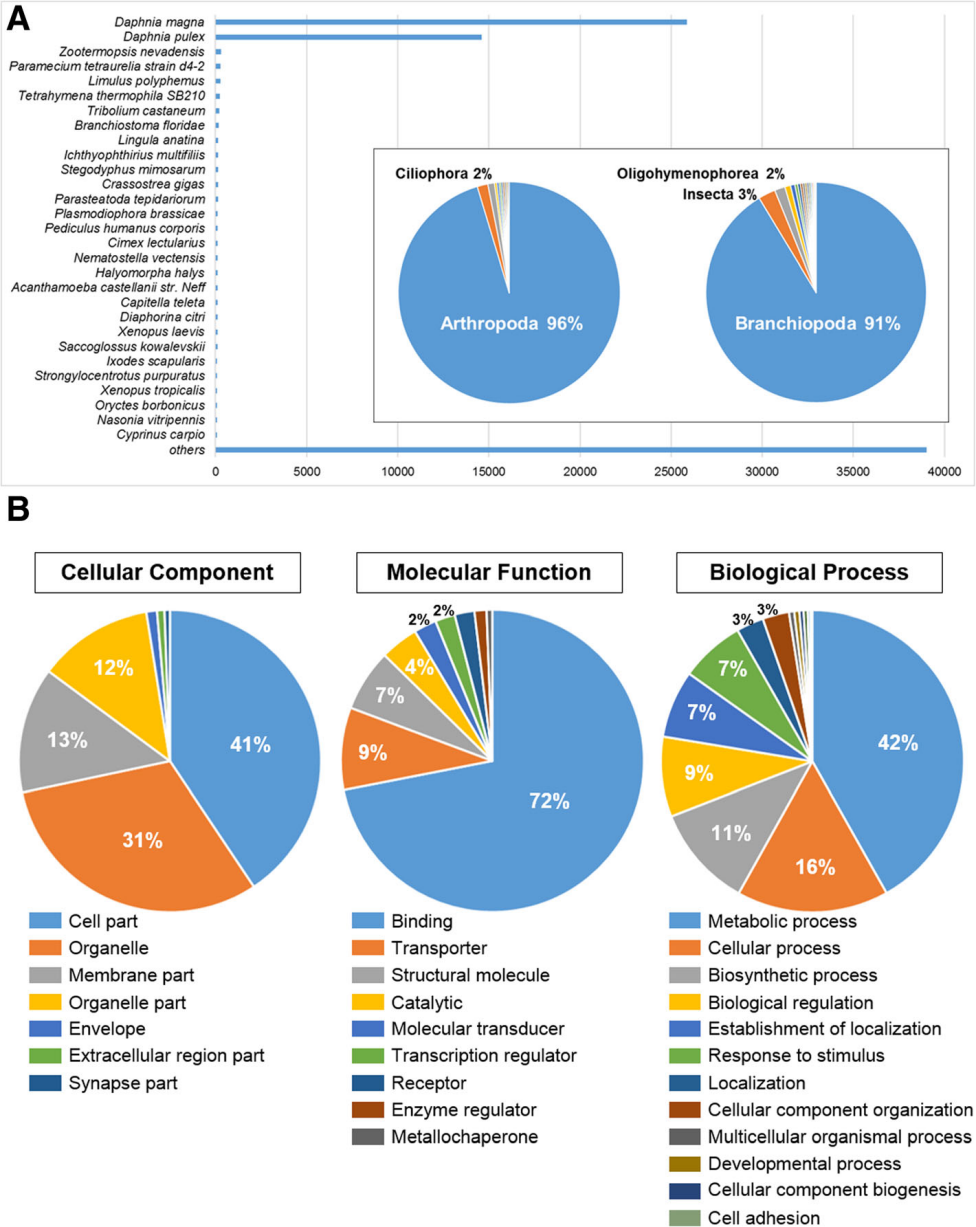
**a** Numbers of major BLAST hits matched to *Diaphanosoma celebensis* transcripts at phylum and species levels. Each number is the number of orthologous gene families shared by the indicated genomic database. **b** Gene Ontology (GO) analyses: cellular components, molecular functions, and biological processes enriched in the *D. celebensis* transcriptome

The aim of this study was to elucidate the potential advantages, particularly in monitoring estuaries and coastal regions, of transcriptional profiling of the brackish water flea *D. celebensis*. Based on principal BLAST hits, about 25,881 *D. celebensis* contigs exhibited sequence similarities to *Daphnia magna* transcripts, and 14,604 to *Daphnia pulex* transcripts (Fig. 1a). 96 and 91% were homologous to transcripts from phylum Arthropoda and class Branchiopoda, respectively. Thus, sample preparation and sequencing had been successful; the raw read assembly was undoubtedly Cladocera. Our reference transcriptome of the brackish water flea will be valuable for studying comparative genomics in Cladocera and facilitate biomarker development for ecotoxicology studies as a sentinel species for estuaries and coastal regions.

Diverse GO assignments analyzed by Blast2GO showed that *D. celebensis* performs several complex biological functions (Fig. 1b). In cellular components category, transcripts were assigned to cellular part (41%), organelle (31%), membrane part (13%), and organelle part (12%). In molecular function category, genes were categorized as binding (72%), transporter (9%), and structural molecule (7%). In biological process category, genes were classified as metabolic (42%), cellular (16%) and biosynthetic (11%). KEGG pathways analysis showed that many annotated sequences were involved in various pathways.

**Fig. 2:**
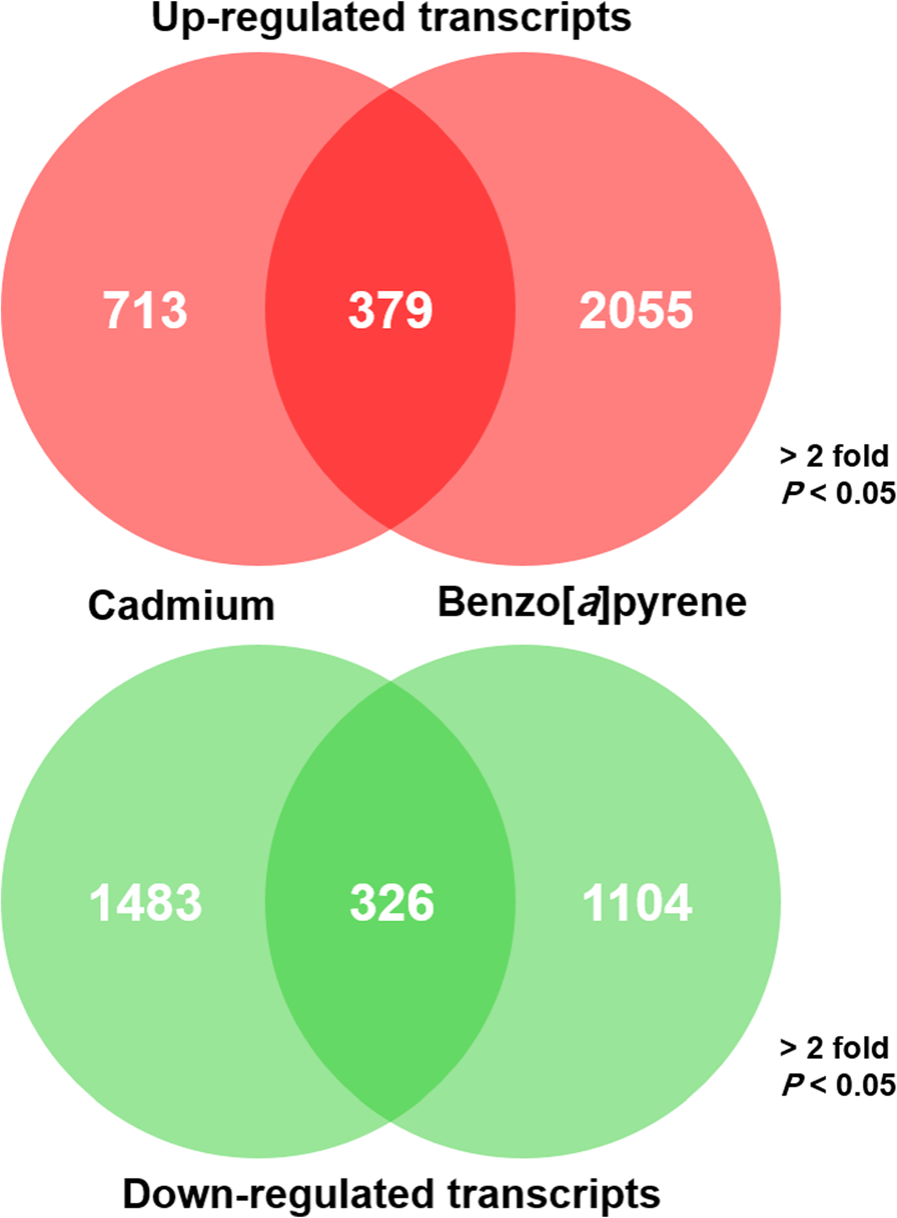
The number of uniquely or commonly up- or downregulated transcripts in the transcriptome of *Diaphanosoma celebensis* exposed to Cd or BaP. Van diagrams were constructed with selected transcripts under criteria, > ± 2 fold and *P* < 0.05

A total of 2901 unique genes showed significantly different mRNA expression in Cd-exposed *D. celebensis* criteria > ± 2 fold change and *P* < 0.05 (1092 transcripts upregulated; 1809 downregulated), and 3864 transcripts showed significant differences in the BaP-exposed *D. celebensis* (2434 transcripts upregulated; 1430 downregulated) when compared to respective controls (Fig. 2). Of upregulated transcripts, 713 mRNAs of Cd- and 2055 mRNAs of BaP-exposed *D. celebensis* were chemical-preferentially expressed, whereas 379 mRNAs were commonly upregulated in both chemical exposures. Among downregulated transcripts, 326 mRNAs were common for both chemicals, while 1483 and 1104 mRNAs were unique to Cd and BaP, respectively. KEGG analysis also identified numerous enzymes uniquely or common to various metabolic pathways (Additional file 1**:** Table S5).

A comparative analysis of *D. celebensis* transcriptomes was performed to identify transcriptional expressions unique to each chemical exposure. Of up-regulated transcripts by Cd exposure (> 2 fold change and *P* < 0.05), mRNA expressions of vitelline membrane outer layer (VMO) protein (10.45 fold), HSP19.9 (3.48 fold), cytochrome P450 (CYP) 3036a1 (3.45 fold), and glutathione *S*-transferase sigma (GST-S; 2.43 fold) were significant. VMO protein (*vmo1* gene) is essential in maintaining yolk and protecting eggs from microbes [23]. Effects of Cd on vitellogenesis are not fully explored in aquatic crustaceans. However, Cd treatment significantly increased copepods’ vitellogenin (*vtg*) gene through putative metal responsive elements (MREs) of promoter region [24]. Cd induces oxidative stress by generating reactive oxygen species (ROS) in oxygen-consuming organisms. Thus, intracellular protective mechanisms including antioxidant defense system (e.g. GSTs) and stress proteins (e.g. HSPs) [25, 26] can be actively induced by Cd exposure in *D. celebensis*. Although limited information on annotation and potential detoxification functions is available on CYP superfamily members in aquatic invertebrates, members of CYP2/3 families are likely involved in xenobiotics metabolism [27, 28].

Among significantly downregulated transcripts (> − 2 fold change and *P* < 0.05), calcium-activated chloride channel regulator (CLCA, − 7.72 fold), trypsin (− 3.58 fold), alpha-amylase (− 3.21 fold), lipase (− 2.20 fold), and endochitinase (− 2.02 fold) were notable. CLCA regulates calcium activated chloride channels (CaCCs), which is involved in cellular physiology, such as neuronal and cardiac action, muscle contraction, and epithelial secretion [29, 30]. Cadmium can interrupt activation of CaCC [31]. Although function of CLCA and CaCCs are scarcely studied in aquatic invertebrates, it is assumed that Cd may disrupts activation of CaCC by inhibiting CLCA expression, as shown in the present study.

Decrease of several digestive enzymes such as trypsin, alpha-amylase, and lipase in response to Cd can be induced by disruption of cysteine (Cys)-containing digestive enzyme through potential binding of Cd to Cys residue [32]. Chitinase, a target of ecdysteroids, regulates crustacean molt process [33]. Thus, downregulation of endochitinase can be associated with Cd-triggered endocrine fluctuation of *D. celebensis*, but further confirmatory evidence is needed.

BaP exposure significantly upregulated mRNA expressions of microsomal GST (*mgst*; 8.58 fold), DNA repair and recombination protein *rad54* (8.29 fold), GST theta (*gstt*; 5.34 fold), and DNA excision repair protein *ercc-1* (4.64 fold), but its treatment decreased cryptochrome1 (*cry1*; − 8.17 fold) and timeout/timeless-2 (− 7.38 fold). Transcriptional increases of *gst* family are not surprising as their molecular and biochemical involvements as phase II detoxification enzymes have extensively been studied in BaP biotransformation metabolism of aquatic animals. Interestingly, mRNA expressions of two DNA repair-related transcripts, *rad54* and *ercc-1*, was significantly increased by BaP exposure. BaP, a carcinogen, exerts its toxicity as benzo[*a*]pyrene diol epoxide (BPDE) through CYP-mediated biotransformation. BPDE can bind to DNA and produce BPDE-DNA adducts leading to mutation, carcinogenesis, and/or cell death [34]. Thus, upregulation of *rad54* and *ercc-1* can be associated with activation of repair metabolism by BaP-mediated DNA damage. In fact, BaP and/or BPDE exposures have significantly increased DNA repair genes including *ercc1* and *rad54* in vitro and in vivo [35–37]. Interestingly, downregulation of two transcripts, *cry1* and timeout/timeless-2, which are involved in circadian clock metabolism, suggests that *D. celebensis* circadian clock (CC) metabolism can also be influenced by exposure to BaP. Regulation of CC is known to be closely related to xenobiotics’ effects [38]. Interaction between aryl hydrocarbon receptor (AHR) signaling pathway and CC is essential in maintaining homeostasis; disruption of biological clock can induce hypersensitivity to environmental toxicants [39].

In conclusion, library construction and assembly were successfully conducted and the transcriptome covers essential gene repertoire of Cladocera in the present study. Finally, we present the first *D. celebensis* transcriptome and a brief comparison of differentially regulated transcripts upon Cd and BaP exposure. Our results show that the application of transcriptome profiling is a promising testing method to study effects of exposure to environmental pollutants using *D. celebensis*.

## Additional file

Additional file 1: **Table S1.** Summary of the library construction. **Table S2.** Summary of the de novo transcriptome assembly results. **Table S3.** Transcriptional expression analysis. **Table S4.** Gene Ontology (GO) analysis for the three categories. **Table S5.** KEGG pathway analysis for uniquely or commonly up- or downregulated transcripts in the transcriptome of *Diaphanosoma celebensis.* (XLSX 21456 kb)

## Abbreviations

AHR: Aryl hydrocarbon receptor
BaP: Benzo[a]pyrene
BPDE: Benzo[a]pyrene diol epoxide
Cd: cadmium
CLCA: Calcium-activated chloride channel regulator
CYP: Cytochrome P450
GO: Gene ontology
GST: Glutathione S-transferase
HSP: Heat shock protein
KEGG: Kyoto Encyclopedia of Genes and Genomes
PAH: Polycyclic aromatic hydrocarbon
RPKM: Reads per kilobase of transcriptome per million mapped reads
